# MedChemExpress compounds prevent neuraminidase N1 *via* physics- and knowledge-based methods†

**DOI:** 10.1039/d4ra02661f

**Published:** 2024-06-12

**Authors:** Quynh Mai Thai, Trung Hai Nguyen, Huong Thi Thu Phung, Minh Quan Pham, Nguyen Kim Tuyen Pham, Jim-Tong Horng, Son Tung Ngo

**Affiliations:** a Laboratory of Biophysics, Institute for Advanced Study in Technology, Ton Duc Thang University Ho Chi Minh City Vietnam ngosontung@tdtu.edu.vn; b Faculty of Pharmacy, Ton Duc Thang University Ho Chi Minh City Vietnam; c NTT Hi-Tech Institute, Nguyen Tat Thanh University Ho Chi Minh City Vietnam; d Institute of Natural Products Chemistry, Vietnam Academy of Science and Technology Hanoi Vietnam; e Graduate University of Science and Technology, Vietnam Academy of Science and Technology Hanoi Vietnam; f Faculty of Environment, Sai Gon University 273 An Duong Vuong, Ward 3, District 5 Ho Chi Minh City Vietnam; g Department of Biochemistry and Molecular Biology, College of Medicine, Chang Gung University Kweishan Taoyuan Taiwan

## Abstract

Influenza A viruses spread out worldwide, causing several global concerns. Hence, discovering neuraminidase inhibitors to prevent the influenza A virus is of great interest. In this work, a machine learning model was employed to evaluate the ligand-binding affinity of *ca.* 10 000 compounds from the MedChemExpress (MCE) database for inhibiting neuraminidase. Atomistic simulations, including molecular docking and molecular dynamics simulations, then confirmed the ligand-binding affinity. Furthermore, we clarified the physical insights into the binding process of ligands to neuraminidase. It was found that five compounds, including micronomicin, didesmethyl cariprazine, argatroban, Kgp-IN-1, and AY 9944, are able to inhibit neuraminidase N1 of the influenza A virus. Ten residues, including Glu119, Asp151, Arg152, Trp179, Gln228, Glu277, Glu278, Arg293, Asn295, and Tyr402, may be very important in controlling the ligand-binding process to N1.

## Abstract 2

MedChemExpress compounds prevent neuraminidase N1 *via* physics- and knowledge-based methods.

## Graphical abstract

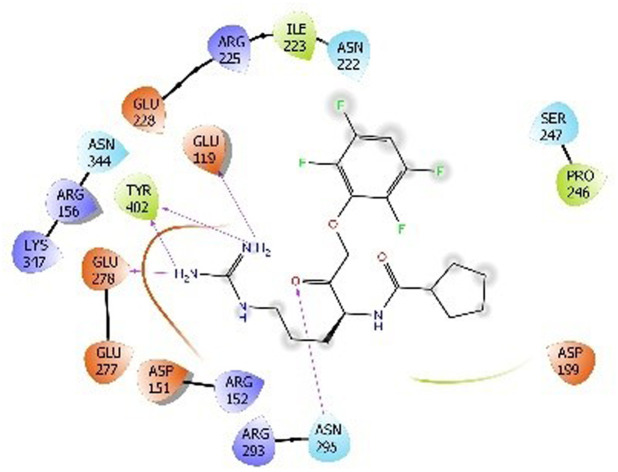

## Introduction

The influenza A virus pandemics killed several millions of people in the last century, including the H1N1 pandemic in 1918, H2N2 pandemic in 1957, and H3N2 pandemic in 1968.^1,2^ In this century, the influenza A virus, including H5N1,^3,4^ H1N1,^5,6^ H5N8,^7^ and H7N9 (ref. 8) caused flu for a large number of people worldwide. Numerous studies were carried out to find a potential inhibitor for treating the disease.^9–15^ In particular, neuraminidase is the most popular drug target since it is an important element in the delivery of viral progeny to human cells.^16^ Oseltamivir, zanamivir, and peramivir are among the drugs approved for inhibiting the target. However, numerous resistances still persist.^17–19^ Therefore, studying new inhibitors that are able to effectively inhibit neuraminidase remains an interesting issue.

Computer-aided drug design (CADD) is a powerful tool for rapidly and accurately screening several million compounds for potential enzyme inhibitors. It has been initially reported since October 5, 1981, when an article entitled “*Next Industrial Revolution: Designing Drugs by Computer at Merck*” was published by Fortune magazine.^20^ CADD's influence is rapidly increasing due to a significant decrease in the cost and time of new drug development.^21^ CADD can be used for both purposes, including searching for new inhibitors and repurposing existing drugs.^22–24^ CADD has been contributing to the discovery of severally available drugs such as dorzolamide,^25,26^ saquinavir, ritonavir, and indinavir.^20^ In CADD, the computational approach is frequently used to probe potential inhibitors that could bind well to a protein target. Thus, determining of ligand-binding free energy is one of the most critical factors in CADD.^27^ Then, researchers developed numerous schemes,^28^ including physics- and knowledge-based methods, to solve this problem.^29–39^ The combination of these approaches may enhance CADD.

In this work, we aim to use a combination of knowledge- and physics-based methods to search for potential inhibitors from the MedChemExpress (MCE) database for inhibiting neuraminidase. In particular, the trained machine learning (ML) model was utilized to rapidly and accurately probe the ligand-binding affinity of *ca.* 10 000 compounds in the MCE database for neuraminidase. We then performed atomistic simulations to confirm the ML results and gain physical insights into the protein–ligand binding process. It should be noted that both docking and LIE calculations were initially validated over eight complexes including 4B7Q,^40^4B7N,^40^4B7J,^40^5NZE,^41^5NZ4,^41^5NWE,^41^5NZF,^41^ and 5NZN.^41^ The critical residues controlling the ligand-binding process of N1 target were probed to clarify the binding mechanism. The structural change of N1 active site was also investigated to understand how ligands effect the enzymic target. Finally, a shortlist of potential candidates emerged. The outcome probably boosts the flu therapy.

## Materials and methods

### Convolutional networks on graphs calculations

The trained ML model used convolutional networks on graphs (GraphConv)^39^ was utilized to search for potential inhibitors for preventing the biological activity of neuraminidase according to unpublished work.^42^ The model was published online at the GitHub URL https://github.com/nguyentrunghai/Neuraminidase/tree/main/ML/code. In particular, the molecular features can be learned on the fly by the deep learning method GraphConv. A molecular graph is distributed to convolutional layers that will learn molecular fingerprints.

### Structure of receptor and ligand

The X-ray diffraction structure of H1N1 influenza virus neuraminidase was downloaded from the Protein Data Bank with the identification of 4B7Q.^40^ The protein structure is complex with zanamivir, illustrating a sophisticated molecular interaction at the active site of the protein. Besides, the ligands were obtained from the MedChemExpress (MCE) database, a comprehensive repository containing information on approximately 10 000 chemical compounds, was utilized as the foundational resource for performing calculations.

### Molecular docking simulations

The modified AutoDock Vina (mVina), which used a set of modified empirical parameters for improving ligand-ranking, was employed to dock inhibitors to the neuraminidase N1 binding site. The docking grid size was set to dimensions of 24 × 24 × 24 Å. We chose exhaustiveness as the default value based on the previous benchmark.^43,44^ The docking modes differ from one another by an amount energy of 7 kcal mol^−1^.

### Molecular dynamics simulations

**Fig. 1:**
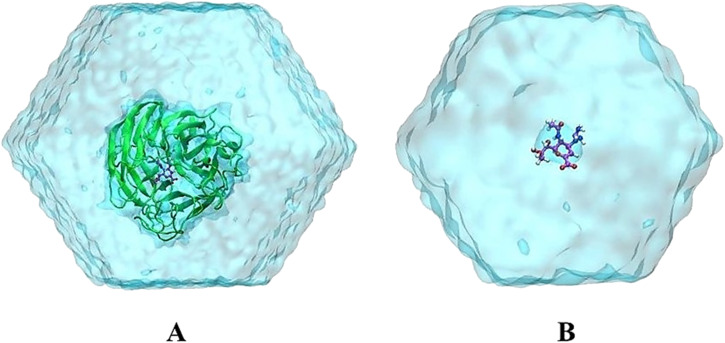
The outset configurations of MD simulations consist of (A) N1 + zanamivir (4B7Q) in solution and (B) free zanamivir in solution. VMD 1.9.3 (ref. 51) was used to provide the figure.

MD simulations were performed to refine the molecular docking outcomes of MCE compounds to neuraminidase *via* GROMACS version 2019.6.^45^ Particularly, neuraminidase and neutralized ions were presented by using the Amber99SB-iLDN force field.^46^ The TIP3P water model was instantaneously used to parameterize water molecules.^47^ Consequently, MCE compound was parameterized using general Amber force field^48^ with the assistance of AmberTools18 (ref. 49) and ACPYPE packages.^50^ It should be denoted that the quantum chemical calculation using B3LYP hybrid functional at the 6-31G(d,p) level of theory was carried out to gain chemical information about the ligand. During which, the restrained electrostatic potential (RESP) approach was employed to allocate the atomic charges over quantum simulations with implicit solvent (*ε* = 78.4).^48^ Moreover, the neuraminidase + ligand complex was placed into a dodecahedron periodic boundary condition (dPBC) box with a volume of *ca.* 570 nm^3^ (see Fig. 1A). The solvated complex comprises 56 000 atoms totally. Besides, the free MCE compound was also inserted into a dPBC box with a volume of *ca.* 66 nm^3^ (see Fig. 1B), which system thus consists of 6500 atoms totally.

The steepest descent method was used to find the lowest energy state for both solvated complexes and free ligands in solution systems. The systems were then relaxed over canonical and isothermal–isobaric ensemble simulations with a length of 100 ps each. The N1 + ligand and free ligand in solution were then simulated over 100.0 and 5.0 ns of MD simulations for each trajectory, respectively. Particularly, the systems were simulated at 310 K and 1 atm.

### Linear interaction energy

Linear interaction energy (LIE) method can be utilized to probe the ligand-binding free energy.^52^ The thermodynamic diagram of the approach is expressed in Fig. S1 of the ESI file.† The ligand-binding free *via* LIE approach, Δ*G*_LIE_, is thus calculated as the average of the van der Waals (vdW) and electrostatic (cou) interaction energy differences of a ligand with their surrounding atoms upon association. In particular, the ligand in the bound state, bound to N1, is annotated as a subscription of b. Besides, the state of free ligand in solution is denoted as a subscription of f. The formula for estimating the ligand-binding free energy *via* LIE method, Δ*G*_LIE_, can be expressed as follow.

1 Δ*G*_LIE_ = *α*(〈*V*^vdW^_l–s_〉_b_ − 〈*V*^vdW^_l–s__f_〉) + *β*(〈*V*^cou^_l–s_〉_b_ − 〈*V*^cou^_l–s__f_〉) + *γ*

whereas the empirical parameters, *α*, *β*, and *γ* can be obtained from the previous works.^53,54^ Among these, the parameters *α* and *β* represent nonpolar and polar interactions, respectively.^53^ The coefficient *γ* is associated with the alteration of the binding cleft hydrophobic mechanism corresponding to various inhibitors.

### Analysis tools

The chemicalize webserver, a tool of ChemAxon, was utilized to predict the ligand protonation states.^55^ The correlation error was calculated using 1000 rounds of the bootstrapping method.^56^ The intermolecular sidechain contact (SC) between the ligand and the residual neuraminidase was counted when the spacing between their non-hydrogen atoms of them is ≤ 4.5 Å. The intermolecular hydrogen bond (HB) between the residual neuraminidase and ligand was counted when the angle ∠ between acceptor-hydrogen-donor is ≥135° and the distance between acceptor and donor is ≤3.5 Å.

## Results and discussion

The trained ML model using the GraphConv technique was used to rapidly and accurately probe the ligand-binding affinity of MCE compounds to neuraminidase N1. It should be noted that the model adopted the correlation coefficient from the corresponding experiments of *R* = 0.80 ± 0.04 according to the unpublished work.^42^ Moreover, the root-mean-square error (RMSE) between ML outcomes and experimental data is of RMSE = 1.86 ± 0.22 referring to the unpublished work.^42^ The ML predicted ligand-binding affinity dropped in the range from −0.06 to −11.12 kcal mol^−1^, with a median of −6.46 kcal mol^−1^. The distribution of the ML predicted ligand-binding free energy of MCE databases is described in Fig. S1 of the ESI file.† Five compounds form an appropriate value of the ligand-binding affinity of smaller than −9.50 kcal mol^−1^ (see Table 1). The atomistic simulations were then performed to simultaneously confirm and explain the ML outcomes.

**Table 1:** Top five compounds suggested by ML model^a^

No.	Compound	Δ*G*_ML_
1	Micronomicin	−10.01
2	Didesmethyl cariprazine	−9.86
3	Argatroban	−9.79
4	Kgp-IN-1	−9.62
5	AY 9944	−9.52

a The unit of energy is kcal mol^−1^.

**Fig. 2:**
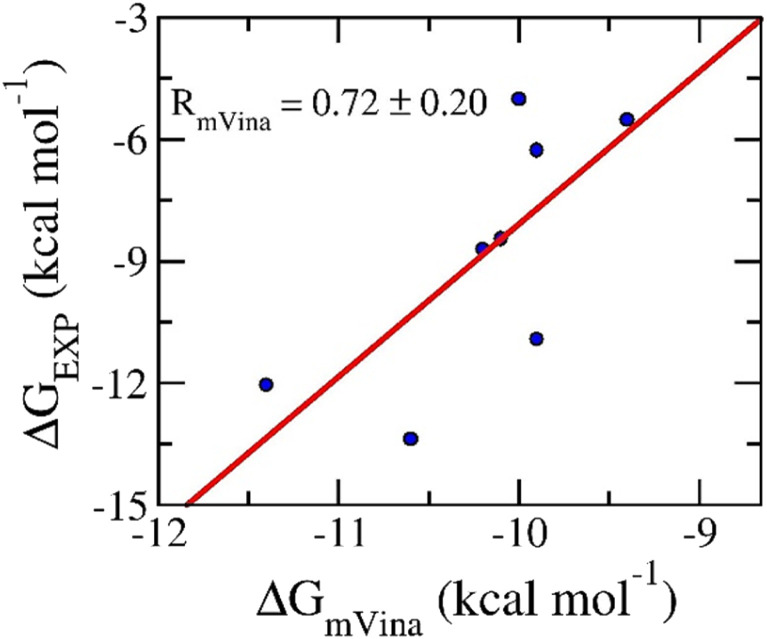
Correlation between docking and experimental data. Docking results were obtained *via* mVina application.

Molecular docking simulations were usually performed to preliminary probe the binding affinities and poses of the ligands to receptors.^57,58^ In this work, AutoDock Vina with the modified empirical parameters^43^ was executed to explore the ligand-binding pose and affinity to N1. Initially, a benchmark was carried out to assess the performance of the docking protocol. The obtained results are described in Table 2. Interestingly, the correlation coefficient between docking and experimental data is of *R*_mVina_ = 0.72 ± 0.20 (*cf.*Fig. 2). Besides, the successful-docking rate, *p̂*, is of 100% since the average of RMSD between docking and experimental poses of RMSD = 1.1 ± 0.1 angstrom (*cf.* Table S1 of the ESI file†). It should be noted that the ligand-binding pose is counted as a successfully docked structure if the RMSD between the docked and experimental shapes is less than 2 angstrom. Therefore, it may be argued that the protocol is an appropriate one to search for ligand-binding pose and affinity for the N1 target.

**Table 2:** The data from docking simulations and experiments of known inhibitors against N1 neuraminidase^a^

No.	PDB ID	RMSD	Δ*G*_mVina_	Δ*G*_EXP_
1	4B7Q ^40^	1.5	−10.6	−13.38
2	4B7N ^40^	1.1	−11.4	−12.05
3	4B7J ^40^	1.3	−9.9	−10.92
4	5NZE ^41^	1.5	−10.2	−8.71
5	5NZ4 (ref. 41)	1.6	−10.1	−8.45
6	5NWE ^41^	1.2	−9.9	−6.27
7	5NZF ^41^	0.8	−9.4	−5.53
8	5NZN ^41^	0.8	−10.0	−5.02

a The units of RMSD, *K*_i_ and energy are Å, nM and kcal mol^−1^ respectively.

**Fig. 3:**
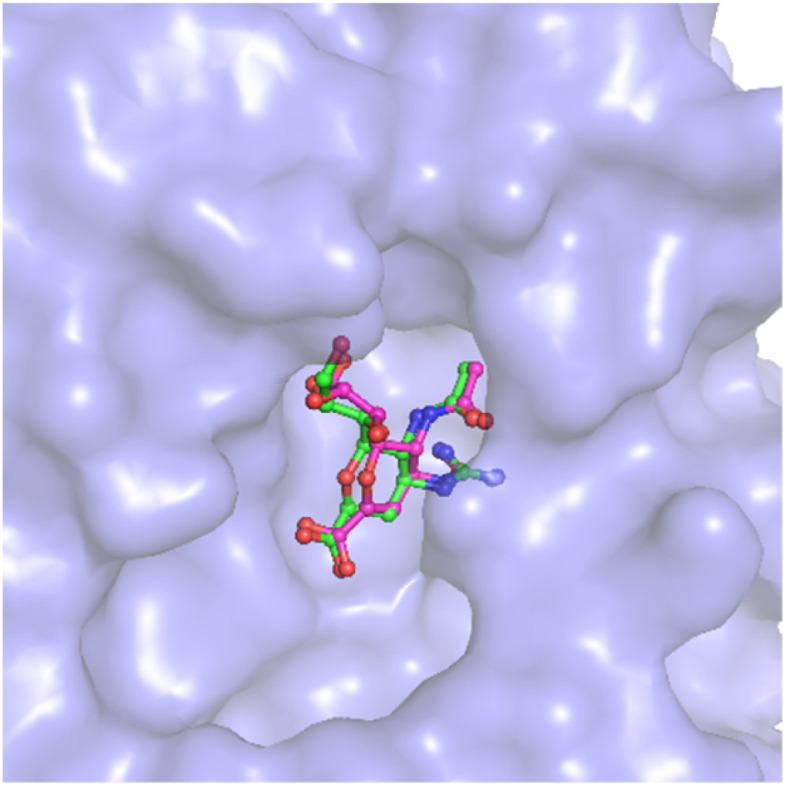
Supper position of docking and experimental pose of the complex 4B7Q. In particular, redocked conformation of zanamivir to N1 was provided by mVina approach.

AutoDock Vina with the altered empirical parameters was thus executed to find the ligand-binding pose of five top-lead compounds to N1. The outcomes of molecular docking simulations were mentioned in Table 3. The ligand-binding free energy adopts in the range from −11.5 to −13.5 kcal mol^−1^, with a median of −12.3 kcal mol^−1^. Consequently, the binding poses of five ligands to N1 are shown in Fig. 3 and Table S2 of the ESI file.† The residues, including Glu119, Asp151, Glu228, Glu277, Glu278, Arg293, Asn295 form rigid contacts with five ligands implying that these residues may play an imperative role in the binding process of ligands to N1.

**Table 3:** Docking energy of the top five compounds to N1^a^

No.	Compound	Δ*G*_mVin_ a
1	Micronomicin	−11.6
2	Didesmethyl cariprazine	−11.5
3	Argatroban	−13.5
4	Kgp-IN-1	−12.8
5	AY 9944	−12.1

a The unit of energy is kcal mol^−1^.

As mentioned above, the molecular docking simulations provided an appropriate outcome. However, because the protocol uses many constrains resulting in a decreasing accuracy of the outcome, MD simulations were thus required to be performed to refine the results. Each MD simulation with a length of 100 ns was carried out to turn the N1 + inhibitor complex into relaxed states, in which the docked conformations were employed as the initial structure of MD simulations. All systems reach the state of stability after 10 ns of MD simulations. Therefore, the structures of the complexes over stable intervals, 50–100 ns (*cf.* Table S3 of the ESI file†), were then used for estimating the free energy difference of binding *via* LIE approach.^59^ Besides, the free ligands in solution were also mimicked over 5.0 ns of MD simulations. The snapshots extracted over an interval 2.5–5.0 ns, which is a stable domain, were utilized for calculating the ligand-binding free energy *via* LIE protocol.^59^

**Fig. 4:**
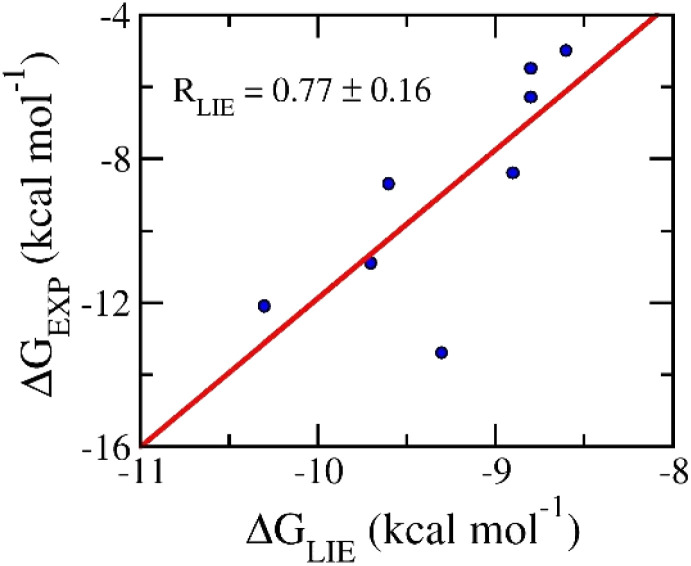
Correlation between LIE and experimental data. In particular, LIE results were obtained over MD simulations.

The ligand-binding free energy between ligands and N1 can be investigated *via* the LIE approach.^52^ In particular, the different free energy between two states involving *bound* and *unbound* states (Fig. S1 of the ESI file†) can be calculated *via* MD simulations by using eqn (1). In conventional, the empirical parameters *α* = 0.18, *β* = 0.50, and *γ* = 0.00 were normally used.^60,61^ Unfortunately, the computational values do not form any correlation to the respective experiments with a value of *R* = −0.74 (*cf.* Table S4 of the ESI file†). Using the different set of empirical parameters involving *α* = 0.288, *β* = −0.049, and *γ* = −5.880,^54^ the LIE outcomes provide an appropriate with the correlation coefficient of *R*_LIE_ = 0.77 ± 0.16 (*cf.*Fig. 4 and Table S4 of the ESI file†). The obtained results imply that the physical insights into the binding process of ligands to N1 are possibility similar to Aβ systems,^54^ Severe acute respiratory syndrome corona virus 2 (SARS-CoV-2) main protease,^62,63^ and Monkeypox virus (MPVX) methyltransferase VP39.^57^ The average of LIE results is of Δ*G*_LIE_ = −9.78 ± 0.25 kcal mol^−1^ that is slightly overestimated for experiments, Δ*G*_EXP_ = −9.26 ± 0.20 kcal mol^−1^. Overall, one could argue that the LIE approach serves as a suitable protocol for estimating the ligand-binding free energy of N1.

**Fig. 5:**
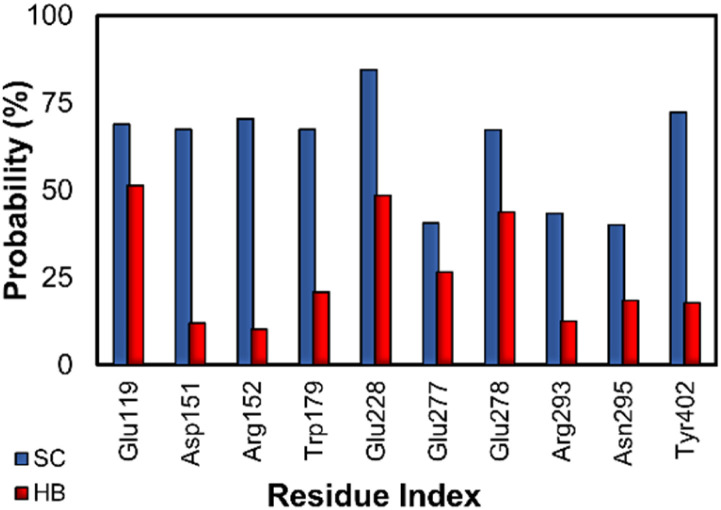
Essential residues forming SC and HB for the inhibitors. In particular, the probability of SC and HB contacts were calculated over an interval 50–100 ns of MD simulations.

The ligand-docking pose of five top-lead compounds to N1 was used as the starting structure of MD simulations. The complex was relaxed over 100 ns of MD simulations, which were also repeated two times. Based on the MD simulation results, the binding mechanism of N1 + inhibitors can be estimated by analyzing the intermolecular HB and SC contacts between N1 residues and their ligands. In this context, the intermolecular HB and SC contacts between N1 and their ligands were calculated over the relaxed intervals of MD simulations. The outcome of the analysis was fully reported in Fig. S2 of the ESI file.† The N1 essential residues, which rigidly form strong HB and SC contacts with inhibitors, are described in Fig. 5. These residues are Glu119, Asp151, Arg152, Trp179, Gln228, Glu277, Glu278, Arg293, Asn295, and Tyr402. On average, the HB and SC contacts between these residues and ligands occupied more than 57 and 29%, respectively. One could argue that possible mutations at these residues can significantly alter the ligand-binding affinity.

**Fig. 6:**
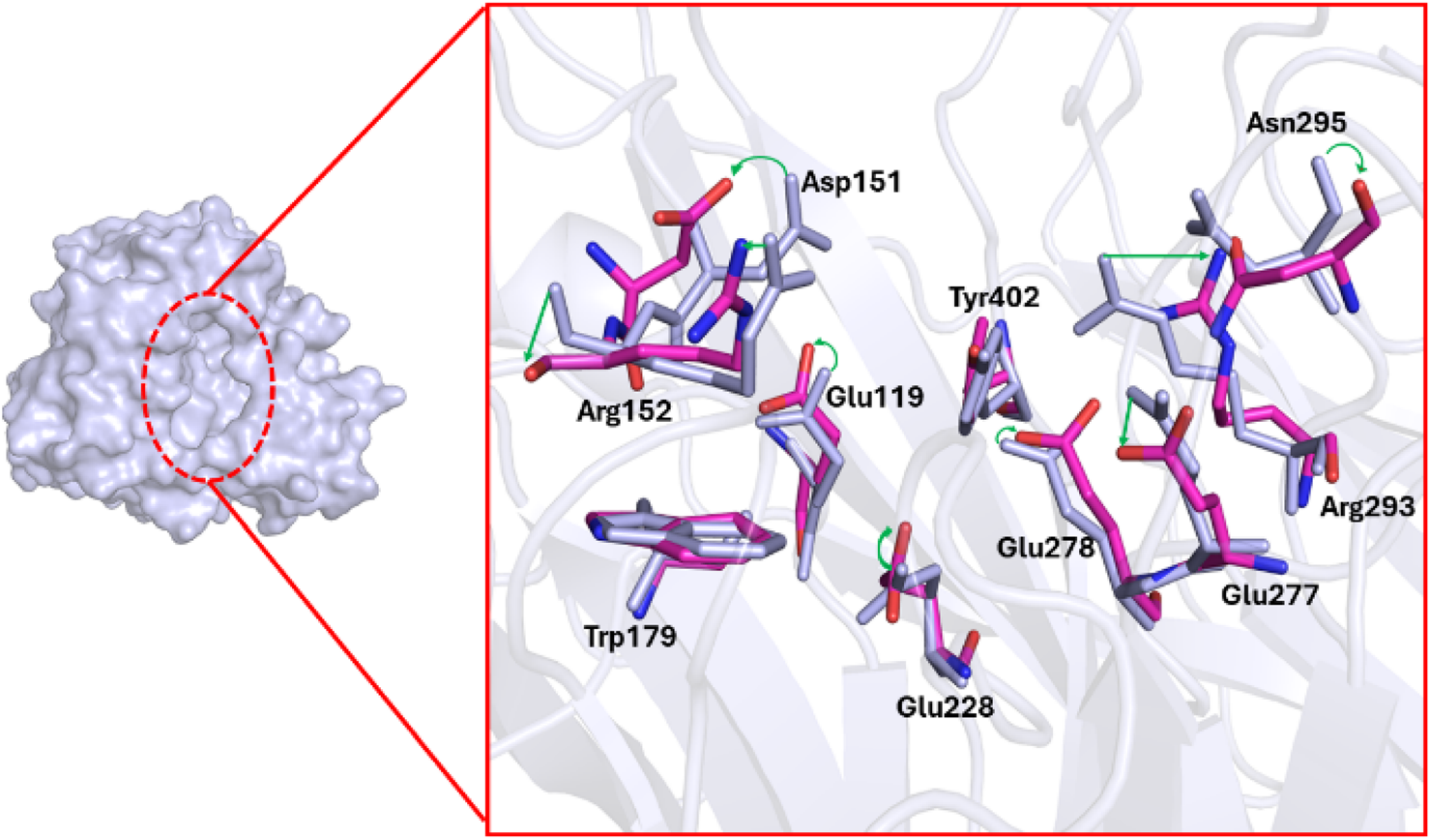
The representative conformation of 10 crucial residues was determined through non-hydrogen RMSD clustering analysis with a cutoff of 1.2 Å. The colorful residues indicate the MD refined conformation compared with the initial structure, which is painted in gray. Changes in the residues are marked by green arrows.

The structural change in the N1 binding site under the effects of their ligands was also probed over the equilibrium conformations of the solvated complex *via* MD simulations. The conformational change of ten essential residues including Glu119, Asp151, Arg152, Trp179, Gln228, Glu277, Glu278, Arg293, Asn295, and Tyr402 was probed *via* the clustering calculation with a non-hydrogen RMSD cutoff 0.12 nm. Fig. 6 represents the superposition between MD-refined conformational and starting shapes, which are noted with colorful and gray colors, respectively. In particular, the green arrows mention the structural change of the corresponding residues. The alteration of the N1 active site possibly implies that the neuraminidase biological activity would be inhibited.

The equilibrium snapshots of the complex over an interval 50–100 ns were used for computing the cou and vdW free interaction energies between ligands and surrounding atoms. Moreover, the free ligand in solution was also simulated to estimate the ligand-binding free energy. The difference in free interaction energies between bound and unbound states can be used as a major factor for computing the ligand-binding free energy *via* LIE scheme. The outcome was presented in Table 4. In particular, the LIE approach is in good agreement with the ML outcome due to the RMSE between ML and LIE results is of 0.70 kcal mol^−1^. On average two computational approaches involving ML and ML, the predicted inhibition constant, Δ*G*, of five top-lead MCE compounds thus range from sub-micromolar to high-nanomolar (*cf.*Table 4). It may thus be argued that top-lead MCE compounds can inhibit the biological activity of N1.

**Table 4:** LIE data of five top-lead MCE compounds to neuraminidase^a^

No.	Name	〈*V*^cou^_l–s_〉_b_ − 〈*V*^cou^_l–s_〉_u_	〈*V*^vdW^_l–s_〉_b_ − 〈*V*^vdW^_l–s_〉_u_	Δ*G*_LIE_ a	〈Δ*G*〉	Predicted *K*_i_ range
1	Micronomicin	−9.05	−12.42	−9.01 ± 0.46	−9.51	Sub-micromolar
2	Didesmethyl cariprazine	−0.52	−13.17	−9.65 ± 0.19	−9.76	High-nanomolar
3	Argatroban	−23.37	−17.07	−9.65 ± 0.63	−9.72	High-nanomolar
4	Kgp-IN-1	−7.47	−18.23	−10.76 ± 0.92	−10.19	High-nanomolar
5	AY 9944	−4.55	−14.40	−9.80 ± 0.87	−9.66	High-nanomolar

a The computed error of LIE approach is the standard error of the mean. The unit of energy is of kcal mol^−1^.

## Conclusions

In this context, physics- and knowledge-based approaches were employed to find potential inhibitors for prohibiting neuraminidase N1 from the MCE database. In particular, the ML model was tested that it formed a good correlation to experiment, *R* = 0.80 ± 0.04, according to the unpublished work.^42^ Moreover, the molecular docking and LIE calculations were indicated to be in good agreement with the corresponding experiments with correlation coefficients of *R*_mVina_ = 0.72 ± 0.20 and *R*_LIE_ = 0.77 ± 0.16, respectively. In particular, the physical insights into the binding process of ligands to N1 are possible similar to Aβ systems,^54^ Severe acute respiratory syndrome corona virus 2 (SARS-CoV-2) main protease,^62,63^ and Monkeypox virus (MPVX) methyltransferase VP39,^57^ because they use the same empirical parameters of LIE approach. In particular, one can be explained since the parameters *γ* associated with the hydrophobic mechanism of the enzymic binding site, while, the parameters *α* and *β* related with nonpolar and polar terms.^53^

Five MCE compounds including micronomicin, didesmethyl cariprazine, argatroban, Kgp-IN-1, and AY 9944 were simultaneously suggested by both physics- and knowledge-based approaches that can inhibit the N1 with predicted inhibition constants ranging from sub-micromolar to high-nanomolar. Furthermore, ten residues including Glu119, Asp151, Arg152, Trp179, Gln228, Glu277, Glu278, Arg293, Asn295, and Tyr402 may play important elements regulating the ligand-binding process to N1. In particular, these residues rigidly form HB and SC contacts with ligands.

## Conflicts of interest

There are no conflicts to declare.

## Supplementary Material
